# PURPOSE AND NUMBER OF CHRONIC HEALTH CONDITIONS AMONG OLDER ADULTS

**DOI:** 10.1093/geroni/igz038.3096

**Published:** 2019-11-08

**Authors:** Janis Sayer, Jennifer L Smith, Cate O’Brien, Joseph G Bihary, Dugan O’Connor, Ajla Basic

**Affiliations:** Mather LifeWays Institute on Aging, Evanston, Illinois, United States

## Abstract

An overwhelming three-quarters of persons age 65 and over have multiple chronic health conditions (Gerteis et al., 2014). With a growing population of older adults, understanding the factors that predict health and reduce the risk of chronic disease is critical. Recent evidence finds that a high sense of purpose- “the belief that one’s life is purposeful and meaningful” (Ryff & Keyes, 1995, p. 720)- is associated with positive health outcomes among older adults. This study investigated the association between purpose and number of chronic conditions among older adults, and whether the relationship depended on age. The study included 6148 older adults (mean age=83.8) who participated in a larger study on wellness. Participants completed a survey that included a measure of sense of purpose and questions about chronic health conditions. Data were analyzed controlling for demographics, optimism, pessimism, social contact, BMI, physical activity, and smoking. Lower levels of purpose were significantly associated with higher numbers of chronic conditions. There was a significant interaction between purpose and age, such that relatively younger older adults with high levels of purpose had fewer chronic conditions. There was no relationship between purpose and number of chronic conditions for the oldest adult participants. The results add new findings to the body of research that demonstrates that sense of purpose is associated with chronic disease. As sense of purpose is modifiable, interventions that increase purpose among older adults, with an emphasis on the youngest-old, should be developed and implemented.

